# Examining venous thromboembolic disease in postoperative neurosurgical and trauma patients in the ICU

**DOI:** 10.1186/cc14405

**Published:** 2015-03-16

**Authors:** D Markopoulou, K Venetsanou, K Venetsanou, E Papadaki, E Papadaki, V Kaldis, V Kaldis, M Mourelatos, M Mourelatos, I Alamanos, I Alamanos

**Affiliations:** 1 KAT Hospital Athens, Kifisia, Greece

## Introduction

Despite preventive anticoagulation therapy measures, venous thromboembolic disease is a major cause of morbidity and mortality among patients hospitalized in ICUs. In fact, pulmonary embolism is not only the most serious manifestation of the disease, but also one of the primary causes of sudden death. The aim of this study is to investigate the frequency of thromboembolism and pulmonary embolism in ICU hospitalized trauma and neurosurgical patients.

## Methods

One hundred ICU patients, 51 postoperative neurosurgical and 49 trauma, were included in the study. Patients' demographic data as well as medical history, temperature, white blood cells and platelets counts were recorded on admission, the day of thrombosis diagnosis and the final outcome of their treatment. Statistics were performed with SPSS-19. *P *< 0.05 was considered significant.

## Results

Thirty-eight out of 100 patients presented thrombosis, 14 trauma and 24 neurosurgical. We examined the correlation of thrombosis development during hospitalization with diagnosis, treatment allocated time and overall patient outcome. It was found that neurosurgical patients developed thrombosis more frequently than trauma patients (*P *< 0.05). In relation to diagnosis, thrombosis was prevalent among patients with brain lesions (*P *= 0.018). Regarding the type of thrombosis, pulmonary embolism was also commonly apparent among individuals with brain lesion (*P *= 0.020). In addition, there was a statistically significant correlation in thrombosis occurrence between hospitalization day (*P *< 0.01) and patients' outcome on discharge (*P *< 0.001). The type of thrombosis was directly associated with poor outcome, especially one that resulted from central catheters (*P *< 0.001) and pulmonary embolism (*P *< 0.001). However, no correlations were found with temperature, white blood cells and platelet counts on admission (*P >*0.05).

## Conclusion

Thrombosis affects the ICU patient's final outcome. The type of thrombosis contributes to a poor outcome and mainly the occurrence of pulmonary embolism significantly increases the mortality rate.

